# ‘Immune from the germ-laden things’: Immunity and Irish Newspaper Advertising, 1890–1940

**DOI:** 10.1093/shm/hkae035

**Published:** 2024-06-20

**Authors:** Maebh Long

**Affiliations:** English Programme, University of Waikato, Hamilton, New Zealand

**Keywords:** immunity, advertising, newspapers, Ireland, immunology

## Abstract

From 1890, as advertising in Irish newspapers grew in quantity and sophistication, a discourse of immunity began to circulate. Advertisers drew on advancements in bacteriology and immunology to present their goods as defensive strategies against a range of threats, from major infectious diseases to everyday coughs and colds. Consumers were urged to supplement their bodies’ vulnerabilities by purchasing pills and tonics, with medical products joined by immunity-assuring underwear, coats, cosmetics and cars. From a dataset of every immunity-focused advertisement in the Irish Newspaper Archives and *The Irish Times* archives between 1890 and 1940, I unpack the ways immunity was presented to the Irish public outside of medical institutions. I show how discourses of immunity intersected with influenza outbreaks, consider the implication of the non-national origins of many advertisements, and trace their rhetoric of protection and resistance across a range of product types.

When William Boxwell gave the inaugural address at Dublin’s Meath Hospital at the opening of the 1912–1913 academic year, he told the listening students that although ‘the most brilliant minds the world over [are] now focussed upon the whole question of immunity’,^1^ it ‘still abides in the main an inscrutable mystery’.^2^ A few years later, in *The Dublin Journal of Medical Science*, Richard Wellington Shegog echoed both Boxwell’s enthusiasm and his concerns, describing immunity as ‘one of the greatest’ of the sciences while lamenting that it ‘raises a thousand questions for every one it even attempts to solve’.^3^ Yet, in Ireland in the same year, products from influenza preventatives to hernia trusses, baby formula to bicycles were marketed through sales pitches promising consumers immunity to a wide range of illnesses and discomforts. The medical profession might have been sobered by the enormity of research needed in bacteriology and immunology, but advertisers in Irish newspapers clearly found the concept of medical immunity sufficiently persuasive to incorporate it into their marketing. The copy for Formamint tablets told readers that with just a few lozenges they could be ‘practically immune’ to colds and sore throats.^4^ Crosby’s Cough Elixir promised to make users so healthy that the ‘chronic sufferer’ could go through the ‘hardest winter with perfect immunity from further attack’,^5^ while Germolene ointment assured consumers that its aseptic action would render a wound ‘immune from poisoning and dirt’.^6^ Immunity was on Irish shelves, ready to give the public a life exempt from disease and difficulty.

In the late nineteenth century ‘immunity’ was a word in transition. When Edward Jenner used cowpox to vaccinate against smallpox in the late eighteenth and early nineteenth centuries, he advanced a process of artificial medical resistance that would gather pace from studies of bacteriology in the 1870s and become established through Louis Pasteur’s work on acquired immunity in chicken cholera in the 1880s. From here theories and discoveries advanced rapidly, with Élie Metchnikoff proposing a cellular theory of immunology in 1884, Emil van Behring and Kitasato Shibasaburō’s publishing their findings on diphtheria antitoxin in 1890, and Paul Ehrlich’s proposing his side-chain theory of antibody formation in 1897. Searching for a word to name the state of being spared sickness, scientists had turned to ‘immunity’, borrowing a term that had originated in ancient Roman law and had gradually came to mean exemption from onerous duties.^7^ Through scientists’ adoption of the term, and by growing public awareness of changes in medicine, discourses of immunity, now predominantly naming an exemption from infection and disease, began to proliferate in popular discourse.

This burgeoning adoption of the medical register of immunity in Ireland took place against the ‘mixed medical economy’ that was operative in the late nineteenth and early twentieth centuries.^8^ During this period, the public drew on a variety of the elements of modern and traditional medicine to cure and prevent illness.^9^ Some still incorporated miasma into their understanding of disease transmission, while in other areas, as recounted by district nurse Bridget Hedderman in 1917, ‘Pasteur himself would fail in teaching the “germ theory”’, because people could not understand how germs ‘lived in the air, in food, and in water, and most singularly resented [their bodies] being singled out as an abode for these minute “foreigners”’.^10^ Conditions and health care provisions also varied greatly across the country, particularly between urban and rural areas.^11^ Despite this uneven, blended scene, companies marketing products made in and outside of Ireland drew on the language of bacteriology and immunology to persuade the public to purchase their goods.

Patent medicine and cure-alls were an important and accessible part of public responses to health concerns in Ireland, and many of the commodities analysed in this article fall into this category.^12^ The language of patent medicine advertising was hyperbolic and frequently underpinned by impossible guarantees, and has long been studied in terms of its falsehoods.^13^ This article is not specifically focussed on the truthfulness or otherwise of the claims made in these advertisements, although it should be noted that both Lori Loeb and Caitríona Foley have argued that the content of many patent remedies, and the broad dietary and lifestyle recommendations they made, were frequently in line with best medical practise at the time.^14^ Instead, this article focuses on the way the advertisements employed versions—sometimes factual, sometimes fantastical—of the language and discoveries of immunology.^15^ It shows that as immunology developed as a field, an advertising language of immunity developed in parallel, drawing on immunology’s changing theories and advances to create a fluid register of risk and risk reduction. By examining the affective and scientific range of the language through which medical and medical-adjacent products were sold in English-language Irish newspapers between 1890 and 1940, we can gain significant insights into the ways immunity was presented to the Irish public outside of doctors’ offices and hospitals. We can consider the implications of the predominately non-national origin of the advertisements on discourses of immunity, that is, on discourses of protection, prevention, exemption and separation. We are also afforded new understandings of the nature of advertising copy in Irish newspapers in the late nineteenth and early twentieth centuries. By employing a digital humanities approach to medical history, this article offers focussed close readings of individual advertisements chosen as indicative of trends within the large scale that digitised platforms provide: that is, from a survey of every advertisement in the Irish Newspaper Archives and *The Irish Times* archives on ProQuest that mentioned immunity or immune between 1890 and 1940.^16^ The start date of 1890 loosely marks the beginning of public engagement with medical understandings of immunity and their early use in newspaper advertising. Prior to 1890 searches produced extremely limited results for the use of immunity in advertising: the deployment of the term in marketing strongly coincides with the circulation of its medical register, rather than its earlier political meaning. From 1890 we not only see the steady growth of an advertising discourse of immunity, but we as we expand below, experience a period of change in advertising itself. We also see in Ireland during this period a growing focus on preventative medicine and the cause of disease, changing management of public health, increased sanitation, a declining life expectancy in comparison to the USA, England and Wales,^17^ high tuberculosis morbidity,^18^ influenza epidemics and a major global pandemic. These decades also saw labour disputes, civil, international and world wars, partition and the formation of the Free State. The period examined here closes with the beginning of the Second World War to mark both the social and medical changes that this period heralded in, not least the beginning of the antibiotic era and new work on immunology by Frank Macfarlane Burnet. But throughout the difficulties and privations of this time, newspapers were printed and products were advertised, people fell sick and sought cures.

## Context: Advertising and Immunity in Irish Newspapers

Advertising in Ireland in the nineteenth and early twentieth centuries has often been depicted as underdeveloped. Hugh Oram’s *The Advertising Book: The History of Advertising in Ireland* suggests that Irish advertising didn’t begin properly until the 1930s,^19^ and contemporary articles such as ‘The Advertising Problem’ in Sinn Féin’s *Leabhar na hÉireann: Irish Year Book* of 1910 argued that too many Irish businesses conducted themselves in keeping with the belief that ‘advertising does not pay’.^20^ Nonetheless, as Tom Grehan, advertising manager of the *Irish Independent*, wrote in 1911, advertising is ‘not by any means so greatly neglected as some would lead us to believe’, and there were ‘many businessmen in the country who have shown a very apt appreciation of the trade-inducing value of advertising’.^21^ More recent scholarship has aligned itself with Grehan’s view and shows that advertising grew rapidly in the early twentieth century: in 1900 there were only five newspaper advertising agents in Ireland, but by 1922 there were 10 in Dublin alone.^22^ By 1929 advertising could be studied at the Rathmines Technical Institute in Dublin.^23^

Newspaper advertising in Ireland began to increase substantially in quantity and in form, as more newspapers published more advertisements that were frequently bigger, used more graphic design and were increasingly for branded goods. Newspaper sales had also grown: in the 1880s about 75,000 copies per day were sold, but these numbers had increased to over half a million in the 1920s.^24^ Although advertising in Ireland might not have been as indispensable for commercial success as it was in America and Britain, by the early twentieth century advertisements were growing features in Irish newspapers, and consumer culture was an inextricable part of life. As Stephanie Rains puts it, ‘although relatively high Irish poverty levels affected levels of actual consumption, they did not prevent the pervasive imaginative effect of a consumer culture’.^25^ This article analyses the technical aspects of immunity that newspaper advertisements presented to the public, while also tracing the potency of the more fanciful, imaginary aspects of the immune life the advertisements evoke. By looking at medical, medical-adjacent and non-medical products advertised through the language of immunology, this article offers a holistic, expansive engagement with the semantic range of immunity, thereby enabling us insights into the ways immunity and being immune were presented to readers and consumers in Ireland, particularly in the early twentieth century.

Public understanding of immunity was not limited to these advertisements, of course, as the marketing that promised that Reudel Bath Saltrates would make users ‘immune from such ailments as colds, influenza’,^26^ or that ‘ABSOLUTE immunity from danger of [typhoid] and other diseases’^27^ could be acquired through the use of Pasteur Filters, didn’t exist in isolation. Its presence in newspapers was in conjunction with thousands of articles on various aspects of immunology and bacteriology, particularly between 1900 and the 1920s. Headlines proclaimed ‘Immunity from Diphtheria’^28^ and discussed ‘Cancer Research: Rays that Make Persons Immune from Disease’.^29^ Topics ranged from the serious—‘Triumph of Science: Vaccine that Makes Dogs Immune from Distemper’^30^—to the rather more speculative—‘Butchers Are Immune: Meat Dealers, It Is Said, Never Die of Consumption’^31^—to the optimistic—‘Cancer Preventative: Wine Drinkers Held More Immune’.^32^ Over and over again, as one would expect in a country with strong farming communities, conversations about immune varieties of potatoes and ways of rendering cattle immune to disease took place.^33^ Reflections on the use of household and garden goods in protecting against the disease were common, from the efficacy of lavender-scented linen to render homes ‘immune from infectious diseases’^34^ to the power of an onion to keep people ‘practically immune from infection’.^35^ Even articles on everyday domestic medicine regularly engaged with the idea of immunity: one provocatively titled ‘Must we Quarantine the Lips against Love?’ provided a stirring description of the germs the lips could carry, and warned that even if the individual bestowing kisses were immune to the diseases they carry, the person kissed may sicken and die, thus making the kisser ‘guilty of manslaughter in kissing’.^36^ The blend of immunity’s medical and political registers here is potent: the kisser’s immunity from disease does not render them immune from prosecution.

In 1911 the *Anglo-Celt* printed the text of a lecture by Dr J. Clarke on infectious diseases, in which he provided his audience with a useful and accessible description of immunity. One way, he explained, of preventing infection is ‘by the production of immunity or immunisation. That is, by so modifying the condition of the possible recipient so that he becomes insusceptible to the influence of the poison or infection when exposed to it’.^37^ This simple definition captures much of the power immunity had as a marketing pitch: when products promised to make consumers immune, they pledged invulnerability and exemption to the germs that the public had been warned were omnipresent, but also, as we will see, to almost every threat and discomfort. The power of immunity for advertisers was its common representation as a longed-for state of inviolate health and protection: in a short piece on the ‘Pursuit Of Happiness’ in the *Westmeath Independent* in 1928, fifth in the list of elements needed for basic happiness was ‘Immunity from severe physical hindrances, as well as from too great care or anxiety’.^38^

Clarke’s lecture moved from his accessible definition to detailed descriptions of various kinds of immunity and their connections with preventative medicine. In responding to the talk Rev. Dr Comey said that Clarke had succeeded in giving a lecture ‘shorn of all that mysterious phraseology with which medical men love to conceal even the simplest disease’.^39^ This suggests that a technical discourse of immunity and microbes, even a talk that provided a detailed breakdown of immunological processes, was not seen as obscurantist, but information that the public needed to know and of which they could be expected to have some knowledge. This knowledge was supported by articles such as the *Irish Independent*’s ‘Some Unsolved Problems of Medicine’, which took the reader back to Jenner and the basic principles of immunology,^40^ and ‘A Hundred Years’ Progress in Healing and Mending’, which walked readers through microbes and phagocytes, opsonins and anaesthetics and the creation of immunity through anti-toxins.^41^ Early articles on immunity in *The New Ireland Review*, a literary magazine founded in Dublin in 1894 by Thomas A. Finlay, were equally technical. Writing in 1894 that immunity had ‘exercised an extraordinary fascination’ for the last 7 years, Edmond McWeeney explained the difference between acquired immunity and natural immunity, absolute and relative immunity and the various humoral and cellular theories of immunity.^42^ Nor was technical content restricted to English. In the *Connaught Tribune* in 1923 a medical article in Irish engaged with ideas of immunity and helped readers unfamiliar with terms in Irish by providing a glossary, which included sgannán múcarach (mucous membrane), foirleathan ar na daonibh (epidemic), galar-rhaorsacht neamh-ghníomhach (passive immunity) and galar-rhaorsacht ghníomhach (active immunity).^43^

## The Symbolic Power of Immunity

As shown below, there are many examples of advertisements drawing on immunity in medically nuanced, technically detailed ways. Before we engage with immunity on these terms, however, it is important to understand the more symbolic, evocative ways that immunity was presented in these advertisements. At its most foundational, Metchnikoff’s recognition of immunity as a defensive process meant that ‘defence quickly replace[d] healing as medicine’s scientifically approved ethos’,^44^ and so, across these advertisements, immunity is associated with shields and physical barricades. Wincarnis, for example, claimed that their tonic wine ‘overcomes disease, creates reserves of energy, and builds a barrier of immunity against ill-health’.^45^ The concept of shielding and resistance is presented even more directly by Yeast-Vite tonic tablets, whose advertisement featured the brand name embossed on an escutcheon. ‘You cannot shut out disease germs’, the copy insists, as they are pervasive, but ‘you can build up reserve strength in your own body and thus obtain immunity’.^46^ The shield that Yeast-Vite provides, then, is not external but internal, a defensive process that turns the consumer’s body into an impenetrable fortress, enabling them to bring their protection with them so that germs became an irrelevancy. For the rare medical products that weren’t ingested or inhaled, immunity’s shield could take the form of a physical talisman, as it did in the case of the Iodine locket, which promised that it ‘KEEPS YOU IMMUNE FROM COLDS, ‘FLU, and ALL KINDRED AILMENTS’.^47^ This locket was immunity made manifest, a charm for complete protection.

Immunity’s shielding power was frequently presented as a narrative of isolation, where even in the busiest of streets and rooms the careful consumer, dosed with various tonics and pills, was effectively separated and removed from danger. Nostroline Nasal Specific, for example, reminded readers that ‘*Infection* lurks in wait for you in every crowded, hardly ventilated tram or train, church or cinema you enter. Guard against it by always using NOSTROLINE nasal specific, which ensures immunity from all germ attack’.^48^ Similarly, in a large ad in 1929, Wincarnis tonic wine promised readers that they could ‘Face ‘Flu without Fear!’, as Wincarnis, drunk to ensure ‘vital energy’, gives the body absolute protection. The image showed a woman, smiling and warm despite the rain and the crowds behind her, as she is wrapped in fashionable winter clothing and sheltered from the rain by her umbrella. The drops of rain clearly represent the dangers of inclement weather while also making visible the threat of the germs that surround her. But inasmuch as the woman is shielded from the rain by her coat and umbrella, she is separated from germs, and the danger of the crowds behind her, by an immunity facilitated by tonic wines: ‘Take Wincarnis *now* and build up the immunity that glorious health gives’.^49^

**Fig. 1:**
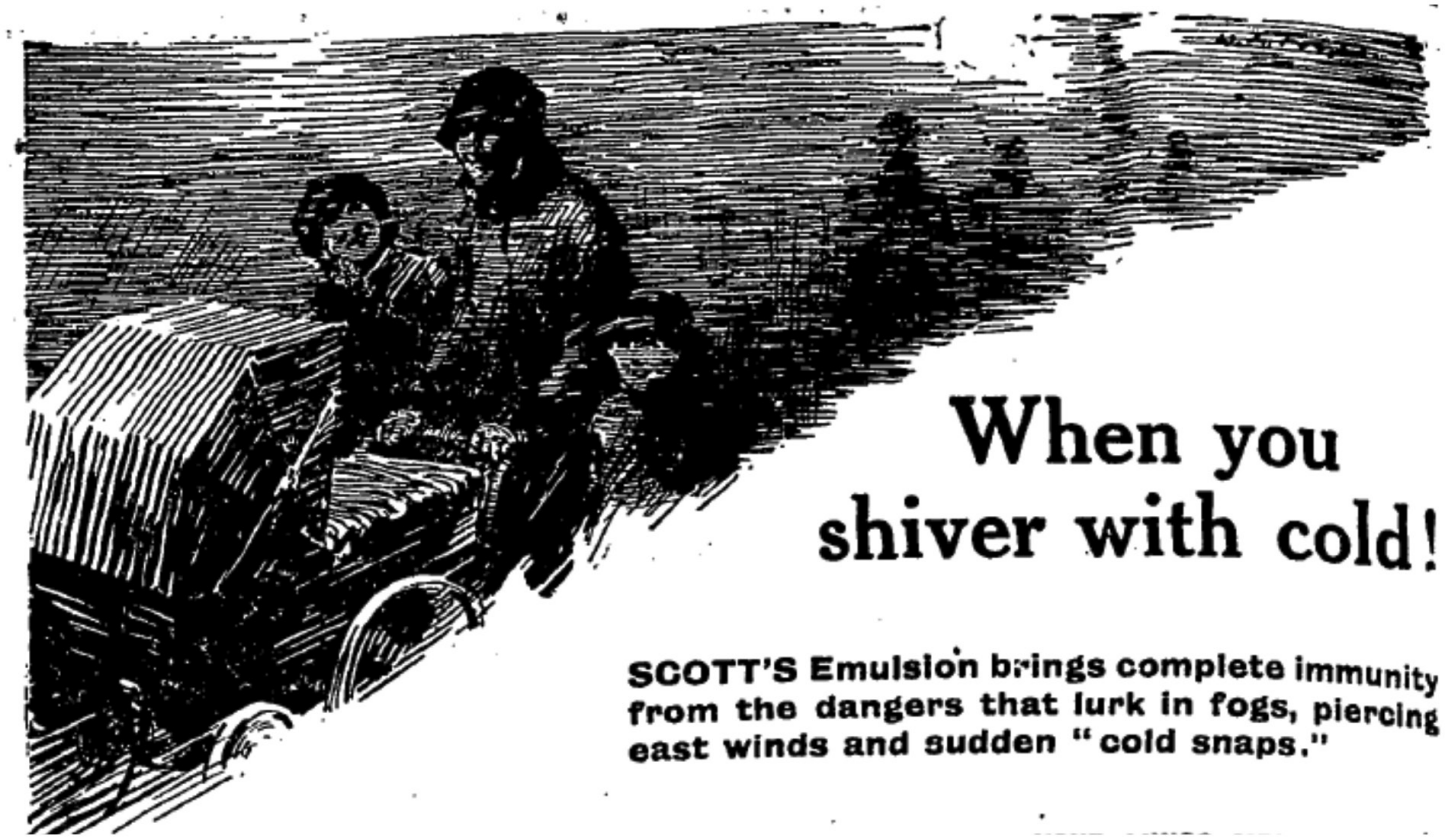
Headline and image of Scott’s Emulsion advertisement, *Leinster Express*, 10 November 1926, 8. Source: Irish Newspaper Archives, www.irishnewspaperarchives.com.

Immunity was almost inevitably used in conjunction with metaphors of menace and strong descriptions of risk, painting a picture of omnipresent threat in order to emphasise commodities’ power of mitigation and protection. Scott’s Emulsion, for example, claimed that their cod liver oil ‘brings *complete immunity from the dangers* that lurk in fogs, piercing east winds and sudden “cold snaps”' (see Figure 1).^50^ Carroll’s Snuff claimed that ‘Thousands and thousands of people have proved that immunity from coughs and colds can be secured during the *danger season* by taking an occasional pinch of snuff’.^51^ Moving from the threat of the weather to the risks of the bacteriological, Hayes Patent Window Ventilator was pitched as a defensive layer that ‘abolishes Dust laden with dangerous microbes, filters and purifies Foul Air, insures immunity from Contagious Diseases’.^52^ Ovaltine advertisements repeated the idea of ‘immunity from attack’ in a series that ran over the 1920s and 1930s, promising that their drink, ‘tired nature’s sweet restorative’,^53^ would keep consumers protected against influenza and later, measles.^54^ Without fail they presented influenza as attacking, either in the form of an epidemic^55^ or because of a bad winter^56^ or sneakily seeming to pass over some only to return when a person’s guard is lowered.^57^ Immunity, then, was a constant defence against an enemy, one that needed to be outsmarted and outgunned at every turn.

Anti-Bi-San, an influenza preventative, took the narrative of protection into the realm of corporate indemnity in the late 1930s. Their product, they insisted, ‘will enable you to enjoy complete immunity from Influenza or your doctor’s bill will be paid by Lloyd’s’.^58^ In a large promotion that included an image of Lloyd’s insurance guarantee, presented on a suitably impressive scroll, these advertisements reminded readers of the prevalence of influenza, the dangers of crowds and the constant risk of infection. Against the threat of sickness, they not only offered ‘complete’ immunity through their pills but claimed that their promise had been underwritten by Lloyds. Immunity, therefore, was ensured and insured: protection was doubled.

The discourse of shielding, isolation, risk and protection was brought together into a compelling picture of comprehensive wellness in promotions for Beecham’s Pills, whose advertisements in Ireland were part of well-funded campaigns the company was running across Britain. These series were lifestyle advertisements selling an existence of total protection and exemption, exemplified in marketing material from 1906 that claimed that ‘happy ones’ who ‘enjoy perfect health […] owe their immunity from illness to BEECHAM’S PILLS’.^59^ Contentment, the copy claimed, that ‘delightful consciousness that all is well with you’,^60^ stems from the perfect protection that Beecham’s provides, thereby rendering health and happiness inseparable. This picture of the pleasures of an immune life, free from all threats, was echoed in a series of advertisements printed the following year, in which the audience was introduced to the ‘“live” man who “gets there”’, the man ‘who never flags and is ever on the “qui vive” [the look out]’. This man, ‘perennially alert and eager’ is ‘cheery, so confident’ not because of a natural state of being, not because he is innately ‘immune from the everyday little ills that upset others so much’, but because he is as ‘prompt in regard to himself as he is in his business’.^61^ That is, this clever, content man has created his immunity by swift, sensible action, an action the advertisements call the readership to replicate. As such, it is not simply pills that are sold, nor simply health that is promised, but an entire life of protection and ease, created by care, diligence and the right medications. This promise was made even more pronounced in 1909 when advertisements argued that the ‘bed-rock’ of happiness that is created by Beecham’s Pills means that those who take it become ‘immune to ill-luck’.^62^ Misfortune becomes a microbe that can be permanently defeated with the correct medication.

The next round of Beecham’s Pills advertisements that furthered the avatar of the immune life—one insulated from harm of every kind—were issued in 1915. These argued that the person of ‘tact and discernment’ recognised when action was needed, and in particular when nature required assistance, as ‘not even the strong and robust are immune from those various aliments which arise out of irregularities of the digestive system’.^63^ Once again the advertisements alluded to the potency of acquired immunity, with Beecham’s Pills becoming the medical intervention the body needed to become immune, not just to ill-health, but to any challenges that could arise. As such, Beecham’s campaigns were selling the desire for generalised security. And they would do this openly in an advertisement that ran in the *Fermanagh Times* between June and September under the headline ‘Security’.^64^ Security in the realm of health, according to the copy, comes from Beecham’s, as ‘great immunity from illness is enjoyed by those who know how to preserve their digestive system, in thoroughly good order’.^65^ But immunity’s function in all these campaigns is always metonymic, that is, even its smaller scale promises—be immune to colds, immune to rheumatism, immune to discomfort—always evoke a larger scale—complete immunity to all ill-health, to all inconveniences, to anything unwanted. The advertisement’s layout, with ‘Security’ as a header and ‘Beecham’s Pills’ as the footer, equates security and Beecham’s pills completely and expansively, without exception, promising thereby to render the consumer absolutely immune.

Significantly, in a number of advertisements the concept of immunity was emphasised to such a degree as to make it a separate, superior category to health. Take, for example, the advertisements for Greene’s Cod Liver Oil that ran in the 1920s and 1930s. ‘KILL THE ‘FLU WITH “BOTTLED SUNSHINE”’, the copy urged, ‘GET A BOTTLE TODAY AND MAKE YOURSELF IMMUNE’.^66^ In becoming immune and entering, so to speak, an all-encompassing, sublime state of total exception, the consumer attains an additional, vital category of complete wellness, signified by the distinctions drawn between health and immunity in the following copy. Be ‘healthy, vigorous and immune from many illnesses prevalent’, the reader is urged, find the secret of ‘glowing health in Winter, and […] remarkable immunity from Colds, Coughs, Chills, and subsequent illnesses’.^67^ These lists are not, or are not simply, lists of synonyms, but indications that within the language of advertising, immunity is health protected, health that is iron-clad, health that is invisible to germs and impervious to threat. These advertisements don’t simply promise health, which is what consumers had before they got sick, but the very impossibility of getting sick again. Health plus immunity, which is health elevated. We can see this depiction of immunity echoed in advertisements like those for Bishop’s Varalettes, which were Lithia tablets for gout marketed to those already suffering, and for whom the lure of a future free of pain would have been extremely powerful. These advertisements assured consumers of the availability of ‘complete relief and future immunity from pain’.^68^ Not merely, then, a return to health, but a health now accompanied by immunity: a superior health as it is unwavering and unbreakable. Similarly, Dr Platt’s Rinex, which was sold as a treatment for hay fever and asthma, promised to ‘go to the root of the trouble *at once* and render you immune from further attacks!’,^69^ with a modified version of the advertisements promising an immunity that ‘frees you *absolutely* from further attacks!’^70^ This was future prevention for consumers who had already suffered but now would never suffer again.

## Immunity and Influenza

There were many products that promised direct cures and the swift creation of future immunity against tuberculosis,^71^ diphtheria,^72^ muscular rheumatism,^73^ kidney complaints,^74^ sciatica^75^ and asthma.^76^ Far more common, however, were the assurances of immunity against everyday illnesses such as colds, coughs and influenza, most of which were presented in terms of prevention through a generalised improved health. In the 1930s, for example, Sanatogen assured consumers it could ‘repair worn nerves, strengthen weak blood and give you the extra vitality that will make you immune from most minor ills’.^77^ In associating influenza with ‘everyday’ illnesses I note its ubiquity, following the pattern of association the advertisements themselves used, rather than its harmlessness. The influenza pandemic in the early 1890s hit Ireland hard, and in 1919 the Registrar-General, Sir William Thompson, announced that ‘Since the period of the Great Famine, with its awful attendant horrors of fever and cholera, no disease of any epidemic nature created so much havoc in any one year in Ireland as influenza’.^78^ Although scholarly interest in the 1918/19 pandemic in Ireland has grown only in the last few decades, and although influenza did not appear to evoke the same concerns as notifiable diseases such as smallpox, the influenza pandemic was by no means unremarked upon in Irish national and regional newspapers, nor in public debate.^79^

During the pandemic, advertisements played on public desire for protection and cures—for the shielding of immunity.^80^ Fort Reviver, a non-alcoholic tonic, claimed that if it was taken three times a day it would ‘ensure that vim and vigour to enable you to ward off the attack and render you immune from infectious diseases’.^81^ Fort Reviver was joined by products such as the Gelsemium Pillules offered by a Dublin pharmacy, by Milton, Citron’s Mineral, and Wilson’s Medical Hall Flu Preventative, all of which promised they could provide immunity to influenza. The advertisement for Gelsemium pills, a homeopathic remedy described as a ‘preventative and a cure’, announced its efficacy under the banner ‘IMMUNITY from the INFLUENZA SCOURGE’.^82^ With similar urgency, Milton deployed a headline warning of the ‘Ravages of Influenza Spreading Everywhere. Safeguard yourself’, but reassured consumers that if the product was used as a mouthwash and nasal spray morning and night, users would ‘be immune’.^83^ The implication, of course, is that the immunity promised is against influenza, but the lack of specificity dangles the possibility of a generalised immunity, so desirable during this period of widespread illness and death.

The assurances of complete protection against such a threat seem irresponsible to the extreme, but they were part of a longer tradition of assuring immunity against influenza by medicated wines like Hall’s Wine, Wincarnis and Winox. Unlike Fort Reviver, these tonics had high alcohol contents and although tonic wines were frequently linked to social anxieties about ‘secret drinkers’, particularly regarding women, who, it was thought, could imbibe alcohol under the guise of medicine, tonics’ commercial successes were in no small part due to the popularity of what Thora Hands describes as ‘drinking for health’.^84^ Alcohol itself was often prescribed by doctors as a treatment for influenza, a fact that Fallon and Son’s played upon in 1923 by presenting their Irish Whiskey as a ‘great tonic’ and insisting that ‘If you want to render yourself immune against the ravages of colds and influenza, then take an occasional glass’.^85^

Tonics usually promised that their products aided vitality and helped the blood and nervous system resist infection. While emphasising the supposedly natural goodness of their product, their sales pitches usually insisted that as natural immunity against influenza was impossible, the individual could only become immune through regular use of their tonic, the sole means of ‘giv[ing] warmth and vitality to the body and render[ing] it immune from chills and colds’.^86^ In 1906, for example, the copy for a Hall’s Wine advertisement claimed that as ‘INFLUENZA AND BRONCHITIS [were] STILL RAMPANT’, it ‘would be idle to expect immunity from coughs and influenza colds, with your bodily strength debilitated by the recent trying weather’.^87^ The body needed to be supported, and immunity created, by a supplement that could both relieve and cure: Hall’s Wine. Similarly, under the bold heading ‘WHY INFLUENZA IS SO TERRIBLE’, in 1907 the copy proclaimed that ‘None but the physically sound and robust are immune’,^88^ so readers were encouraged to use Hall’s as a preventative against influenza and, should they have succumbed through a lack of preparation, as a restorative during convalescence.

Foley argues that responses to the 1918/19 pandemic, which included calls for vaccines against influenza, showed that bacteriology and immunology were being gradually accepted by the medical community. She further claims that by the end of the pandemic ‘the vocabulary of bacteriology was also filtering through more extensively into popular discourse’.^89^ Although the language of newspapers and advertising is not the language of the general public, the prevalence of articles and advertisements that mentioned germ theory and immunity show that this dating is rather conservative. Take, for example, a Hall’s Wine advertisement from 1909. Under the triumphant heading ‘The Passing of the Microbe’, the copy claimed that Hall’s Wine ‘has been, for over twenty years, a constant and never-failing source of maintaining the health and stamina of thousands, and thus rendering them immune from attacks of influenza.’^90^ Drawing on a powerful narrative of medical advancement through bacteriology—‘now that the bacilli of so many diseases are known and recognised, we are able to meet them—to combat them—and to render their attacks upon us comparatively harmless’—this long advertisement positioned Hall’s Wine as a modern supplement for a modern approach to health. The next year Hall’s Wine was still drawing strongly on the language of bacteriology, but presented an even broader claim: ‘Let the winds blow, let the air be charged with microbes, let infection surround you as it will, you will be *immune to all dangers* if you have fortified yourself with Hall’s Wine’.^91^ Danger was no longer simply microbial—the kind of immunity that Hall’s Wine could provide was without limits.

The advertisements not only played on the affective, emotive range of immunity’s associations but could impart relatively nuanced understandings of types of immunity. Take, for example, Allenburys, a company who made baby formula. Their advertisements gave indirect instruction on passive immunity when they told readers that the ‘milk of a healthy mother confers a degree of immunity to infection to the young infant’.^92^ A later advertisement seemed, initially, to use immunity in a more standard way, but it too equated its products with the immunity that breastfeeding affords a child. The copy states that during summer the ‘ever present danger of bacterial infection becomes acute’, which makes a mother strive to ensure that ‘her child’s food is rendered immune from all outside sources of contamination’.^93^ The Allenburys ‘Progressive System of Infant Feeding’ can give that reassurance, the copy insists, as it is a ‘complete safeguard’ whose pasteurising processes ensure ‘absolute food purity’. Amidst the language of safeguard and purity, it is significant that the initial claim is that the baby food itself is made immune to germs. As such, the copy implies, when the baby eats Allenburys baby food it is ingesting immunity, as it would have done through the antibodies of breastmilk. Allenbury’s food is thus again presented as a source of passive immunity equal to breastfeeding.^94^ Of course, the desire to sell products would have been a greater motivator than an interest in educating the public, as we see in the misuse of natural immunity in advertisements for rupture locks. A belt for hernia sufferers, this product claimed to bring ‘rapid relief and a natural and permanent immunity from any recurrence of the disorder’.^95^ The emphasis on ‘natural’ immunity is significant here, as it implies that the belt can create complete resistance without artificial intervention. If natural immunity is the resistance the body can acquire once it has been infected, in this advertisement the belt is positioned as enabling the body to recognise the ‘germ’ of a hernia and resist it. Impossible, yes, and a misapplication of immunology, but no doubt persuasive.

A discourse of immunity is tightly bound up with vaccinations and preventative medicine. McWeeney’s *New Ireland Review* article makes an impassioned case for the importance of research on the body’s defensive apparatus, arguing that ‘With the solution of the immunity-problem is bound up the realisation of the medical scientist’s day-dream—the hope that the medicine of the future may be preventative as well as curative—that the physician’s art may enable him to forestall the advancing foe and thus save his patient from the loss of time, of comfort, and of bodily strength consequent upon disease’.^96^ Wellington Shegog described the ‘greatest triumphs’ of immunological research to be in the sphere of preventative medicine but stressed that ‘the individual is always difficult to deal with in matters of prophylaxis. He is never keen to submit to the inconvenience of inoculation against diseases, the reality of which has never been brought home to him by the only proof he understands’^97^—hence, he argues, the neglect of smallpox vaccination in Ireland. Addressing the nineteenth-century context, Deborah Brunton sees this supposed neglect as born of reluctance rather than a committed anti-vaccination sentiment in Ireland.^98^ The Irish Anti-Vaccination League was founded in 1905, and while there were occasional pockets of concern about vaccination, they never achieved the popularity or mobilisation of the anti-vaccination movement in Britain.^99^ As an article in the *Dundalk Democrat* in 1917 argued, with some exasperation, the ‘anti-vaccination craze […] is not Irish. Like a lot of other things, more or less undesirable, it has been imported from England. […] England is the home of cranks—in medicine, in religion, and in a lot of other things’.^100^

**Fig. 2:**
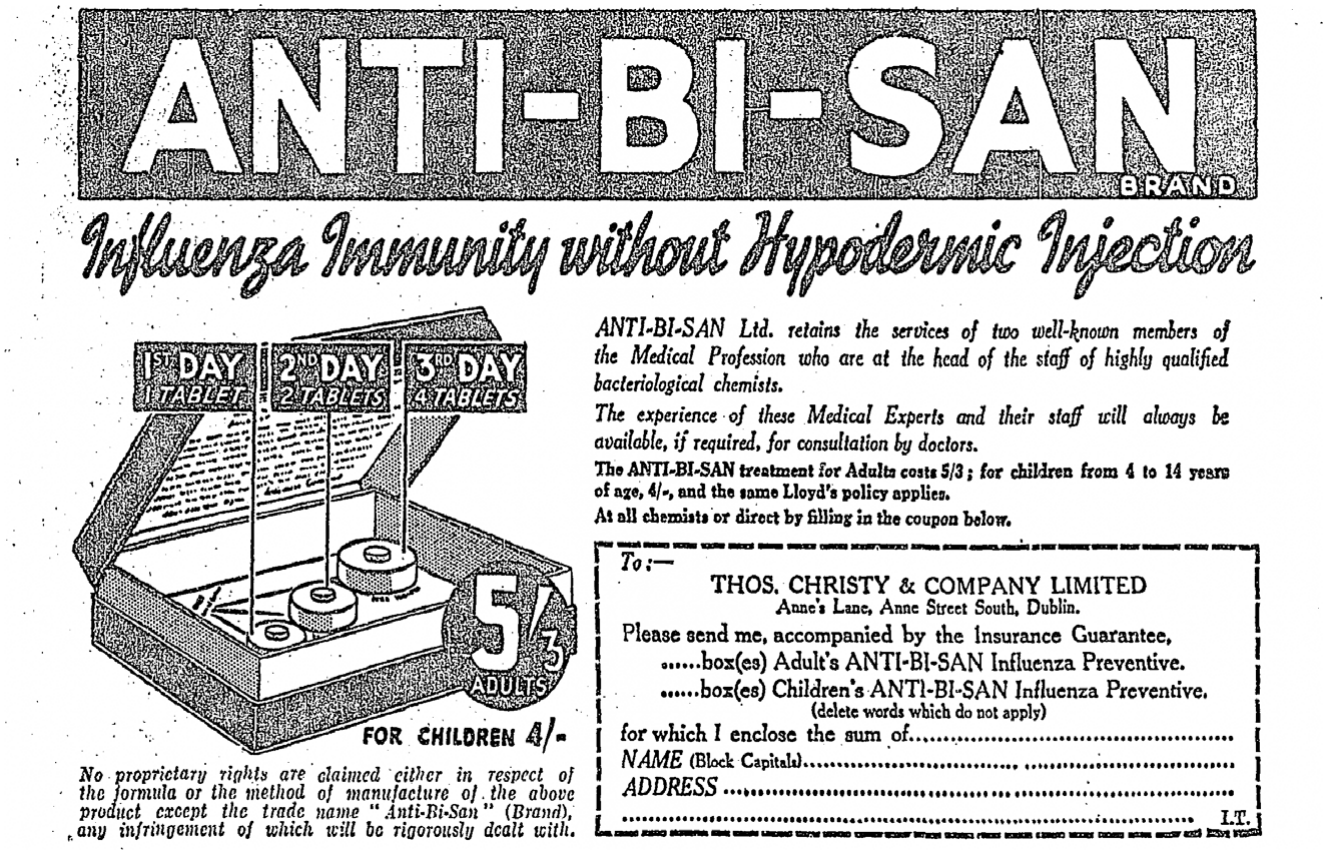
Section of advertisement for Anti-Bi-San, *The Irish Times*, 6 January 1937, 3. Source: *The Irish Times* Newspaper Archive.

Regardless of the public’s strength of feeling about the immunity acquired through vaccination,^101^ many advertisements played on the power of immunisation programmes while presenting their products as a more comfortable, more easily administered mode of rendering the individual immune. They drew on the ability of the vaccine to confer immunity to present their products as having the power of the latter without the inconvenience of the former. The most direct example of this is in the influenza preventative Anti-Bi-San, whose advertisements in the 1930s proclaimed their ability to create ‘Influenza Immunity without Hypodermic Injection’ (see Figure 2).^102^ ‘We are not claiming to cure ‘flu’, they admitted. ‘Science admits quite frankly that she has not one single weapon with which to fight ‘flu germs’.^103^ Instead, they urged readers to take preventative action, and ‘Inoculate yourself against it. Not by injection which is unpleasant, occasionally dangerous, and always costly. But simply by *swallowing* tasteless tablets’. This process, which they term ‘inoculation-by-mouth’, conjures all the power of vaccination without the fear of the needle, and could appeal both to those who supported vaccination and many of those who did not. The desire to create immunity through the least possible intrusion reached its zenith when Odearest Sanitized Mattresses, which were described as ‘antiseptic, self-sterilizing and permanently germ-proof’, claimed that consumers who slept on one would become ‘immune from the dangers of infection’.^104^ No needles, no pills, no vitamin-laden foods: immunity was presented as such an accessible commodity that it could be acquired through sleep.

## Selling Immunity, Not the Nation

Although advertisements using immunity featured in regional and national newspapers, the majority of these advertisements were in national newspapers such as *The Irish Times* and the *Irish Independent*. The *Irish Independent* was a nationalist, Catholic, middle-class paper that sold around 100,000 daily copies in 1915, rising to 140,000 in 1920, a circulation that was greater by tens of thousands each day than *The Irish Times*, a Protestant, Unionist paper.^105^ The majority of the advertisements using a discourse of immunity were for products that were not made in Ireland, nor specifically for an Irish market, and the fact that there was relative parity between the number of advertisements using immunity placed in *The Irish Times* and the *Irish Independent*, despite the fact that the *Irish Independent* had a much higher circulation and a greater focus on advertising, shows a slight marketing bias towards Anglo-Irish consumers. This bias is further evinced by the fact that, despite its much lower circulation, the *Belfast Newsletter* also had a large number of advertisements using immunity. It is also quite clear that the majority of the advertisements made little effort to tailor their copy for an Irish context. Occasionally an advertisement would mention an Irish name^106^ or make a slight adjustment to an image, as Anti-Bi-San did when they changed an image of mocked-up newspaper headings to include ‘Ireland Gets The ‘Flu’.^107^ These were, however, decidedly in the minority. On the whole, advertisements ran with the same copy and visuals they used outside of Ireland. Phosferine, a tonic for joint pain, influenza, nervous disorders and fatigue, placed advertisements promising immunity to various health concerns with testimonials from English concert singers, cricket players and admiralty divers, as well as the American performers, Fred and Adele Astaire.^108^ In 1924 Ovaltine placed advertisements that listed influenza deaths in England and Wales but made no mention of Ireland.^109^ Fort Reviver, a non-alcoholic tonic, reminded readers in 1919 that there had been ‘100,000 deaths in 8 Weeks in this country alone’ from influenza, a statistic complicated by the fact that they ran the same article with the same mortality rate in newspapers in Britain and provided no information on their source.^110^

Not all advertisements came from companies outside of the island of Ireland. Advertising in papers including the *Freeman’s Journal* and *The Irish Times*, one Professor Kennedy promised ‘instant relief and perfect immunity from pain of the most painful swollen or inflamed corn’ from 1893 to 1910,^111^ and Murphy’s Instant Toothache Remedy made similar claims about immunity from pain.^112^ White’s Wafer Oats and Pat Oats, both products made by Belfast companies, insisted that the ‘secret of immunity from Influenza’ lay in the health imparted by a good breakfast.^113^ Auctioneers from across the country made immunity from the disease a popular sales pitch for house and land sales in a wide range of regional and national newspapers.^114^ No farmer or gardener growing potatoes could have been unaware of the need to purchase seed potatoes that were ‘immune from Wart Disease’,^115^ and advertisements placed by Irish companies for immune varieties of potatoes appeared regularly from 1918, with a particular concentration in 1922 and 1923. In Dublin, advertisements for Dockrell’s Paint from 1907 to 1929 promised that their paint rendered ‘exposed surfaces immune against weather ravages’,^116^ and O’Keeffe’s made a selling point of the ‘comfort and immunity from chills which nothing but ALL WOOL will give’.^117^ An advertisement for Greene’s Cod Liver Oil, made by Dublin fishmongers incorporated an Irish heading—‘SLÁINTE NA SAOGHAL AGAT (Health and Long Life to You)’—but theirs was the only instance in which an advertisement used both immunity and Irish.^118^ If immunity was not considered by either Irish or British companies to be a concept unsuitable for marketing in Ireland, it was also clearly not considered to be a word that spoke innately to Ireland or one vital to a nationalist agenda. Yet it could have been, given immunity’s semantic range of medical resistance and political exemption.

A large amount of scholarship has focussed on the connections between narratives of health and the language of conflict,^119^ and as we’ve seen above immunity was associated with attacks and defence. Early advertisements for Bovril, for example, a meat extract paste made in Britain, emphasised the importance of Bovril for soldiers at the Boer War battle of Tugela and claimed that ‘A cup of Hot Bovril, taken now and again, frequently secures entire immunity from [influenza], or, where an attack is imminent, enables the system to resist the scourge with the minimum amount of harm to the body’.^120^ Around the body of the advertisement the phrases ‘BOVRIL AFTER THE BATTLE’ and ‘BOVRIL REPELS INFLUENZA’ were repeated, thereby conjoining the fight undertaken by the soldiers and the fight undertaken by influenza sufferers. The military and the medical coalesce to present the indispensability of defence and resistance, that is, of immunity, for bodies tired by war or civilian life. Yet there was not a rise in conflict-oriented, nor political resistance-oriented, language in the immunity advertisements placed by Irish companies during the War of Independence or the Civil War or the following years of the Free State. The political possibilities embedded in the medical registers of immunity could have made powerful messaging as well as drawn the language of bacteriology and immunology specifically into an Irish sense of self.

## Beyond the Medical

This article has focussed on the advertising of medical products, but it is important to situate these medical advertisements in the context of a wider range of products that were sold through a medicalised discourse of immunity. Readers of the frequent advertisements placed by Joseph Lowry and Sons, Auctioneers, Valuers and Cattle Salesmen, would have been familiar with the term ‘immunity’ as a sales point for land and houses, as between 1890 and 1940 they regularly described fields and livestock as having ‘immunity from disease of any kind’^121^ or simply having ‘immunity from disease’.^122^ Consumers were assured that they could wrap themselves in immunity from the inside out: woollen underwear, for example, promised to ‘keep you warm this Winter, immune from chill and damp’,^123^ while companies selling mackintoshes insisted their special mode of weaving ‘secures immunity from wind or dust and yet affords perfect ventilation’^124^—thus avoiding the unpleasant perspiration often associated with waterproofs.^125^ Even shoe companies promised immunity from disease.^126^ Skin and hair care products were similarly swept up in the language of immunity. Consumers in Ireland could buy Victoria deodorant to attain ‘complete immunity from any recurrence of [the] distressing malady’ of malodorous feet.^127^ Ven-Yusa Shampoo Powder claimed that their powder ‘renders the scalp and hair immune from the germs and other disease carriers that are daily picked up in the factory and shop’,^128^ while Cuticura skin ointment’s ‘unique medical and antiseptic properties ensure immunity against blackheads, pimples, rashes, eruptions, and check the ravages of major troubles such as psoriasis and eczema’.^129^ Broadly speaking, the rhetoric employed in these advertisements indicates the prevalence of anxieties about health and the powerful sales pitch that selling health had become. More specifically, however, in these advertising campaigns we see the extension of a life of absolute protection and exemption from threat. Frequently that threat took the form of germs and microbes, but equally prevalent was the threat of harsh winters and difficult weather: throughout, the language of immunity offered a powerful mode of absolute resistance and complete exemption.

Rather more unexpectedly, bicycles, cars and associated transportation equipment were regularly advertised through promises of immunity. Some quietly made implied connections to medical immunity, like an advertisement for Humber Cycles in 1906, which claimed that their products were ‘IMMUNE FROM ALL ILLS THAT CHEAP BICYCLES ARE HEIR TO’.^130^ Goodrich Tyres, a few years later, expanded upon this wording, still aligning themselves with health-adjacent language: ‘We don’t claim that [our tyres] are immune from these ills that other tyres are heir to, but THEIR CONSTITUTION IS SOUNDER, THEY SUFFER LESS’.^131^ Similarly, an advertisement for the new Tudor Sedan from Ford in 1924 played upon the kind of health concerns nerve tonics guarded against when the copy argued that its ‘luxurious interior and smoothly-running mechanism ensures complete immunity from driving fatigue or nervous tension of any kind’.^132^

**Fig. 3:**
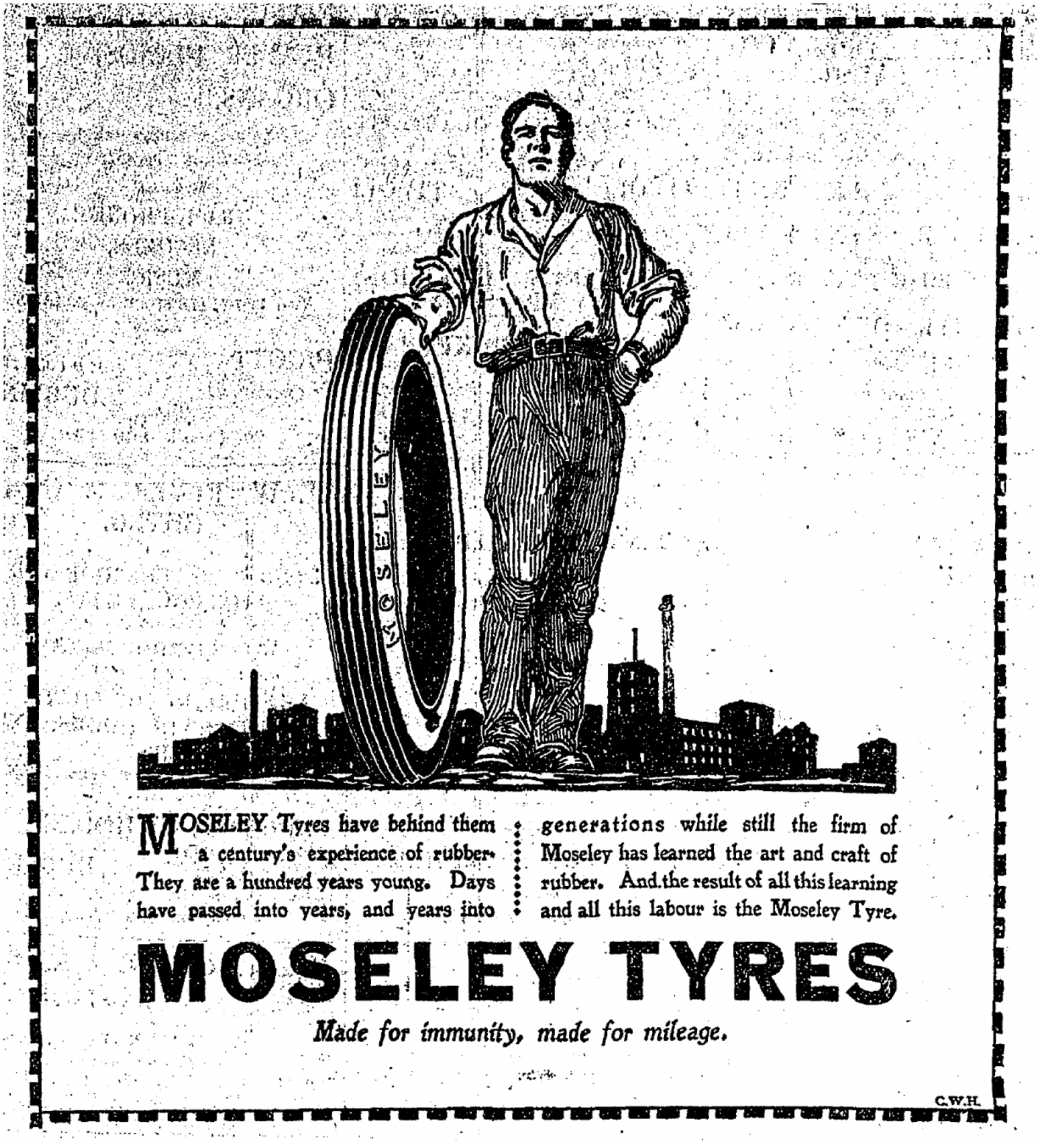
Advertisement for Moseley Tyres, *The Irish Independent*, 22 June 1920, 7. Source: Irish Newspaper Archives, www.irishnewspaperarchives.com.

Not all transport companies felt obliged to spell out the medical association of their use of immunity, however. For these companies the associated power of the word was sufficient: the immunity from trouble^133^ and worry^134^ and risk^135^ and accidents^136^ and danger^137^ that was regularly promised was inseparable from the images of absolute protection and isolation that medical advertising claimed to guarantee. When Mosley Tyres proclaimed in 1920 that their products were ‘Made for immunity. Made for mileage’^138^ it was not a picture of legal exemption that they were creating: they were drawing on the associations of absolute protection and complete security that medical advertising had established and entrenched (see Figure 3). If taking tonics and pills enabled a consumer to walk through germ-ridden crowds unscathed, Citroën and Peugeot enabled a driver to motor Ireland with the same level of protection: their cars were the vehicular manifestation of modern medicine’s—or at least modern medical advertising’s—enveloping preservation. Nothing outside the car could contaminate its stability: it was a mobile immunity that ‘guarante[d] you immunity from *all* danger’.^139^

The language of advertising is not everyday speech, and the public were not naive about the exaggerated claims made in marketing, particularly for cure-alls and patent medicines. Advertising language does, however, still afford us an insight into the rhetoric that advertisers thought would persuade the public to purchase particular goods, and thereby the discourse that surrounded readers and consumers. As Grehan wrote in 1919, ‘the successful advertising man […] must know how to put his thoughts in a clear, convincing sequence, in a way that appeals, that goes straight to the heart of the reader’.^140^ The abundance of advertising means that even if potential consumers rarely read advertisements with focussed, analytic care, the proliferation of these advertisements and their repetition would have gradually reinforced particular ideas of immunity. And some of these ideas were quite peculiar, as they potentially enabled risky behaviour through a narrative of absolute protection from risk. A product named Nic-o-cin, for example, which made the pernicious claim of neutralising nicotine and tobacco tar, evoked the lure of complete resistance to harmful substances by claiming that ‘One Nic-o-cin pastille eaten last thing at night soon gives you complete immunity from the effects of Nicotine poisoning’.^141^ Nic-o-sin presented itself as a means through which the risks of tobacco could be rendered harmless, thereby turning the noxious into an easy pleasure. ‘If all smokers’, they argued, ‘would take the simple precaution of making themselves immune from Nicotine poisoning they could smoke to their hearts’ content without any harmful after effects’.^142^

**Fig. 4:**
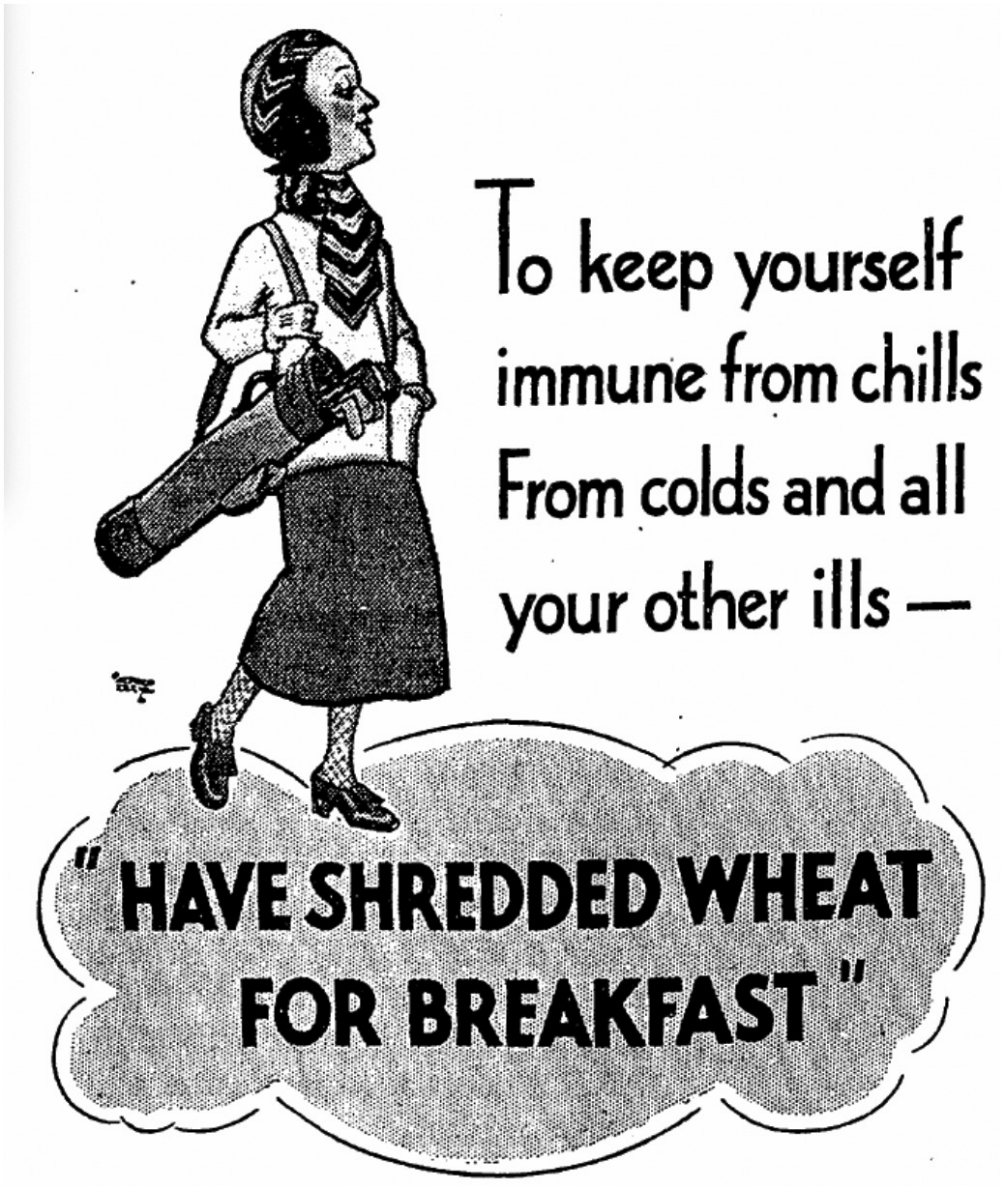
Advertisement for Shredded Wheat, *Irish Press*, 16 December 1932, 2. Source: Irish Newspaper Archives, www.irishnewspaperarchives.com.

The varied health crises of the late twentieth and early twenty-first centuries, including AIDS and COVID-19, have brought new attention to social constructions of immunity.^143^ This article has considered the discourses of immunity sold in newspapers in Ireland, a country regularly overlooked in historical and theoretical engagements with immunity, and brought the narrative back to the early days of public engagement with immune defences, a time frequently less studied within theroetical and philosophical analyses of immunology, thereby providing new insights into the foundations of sociocultural conceptualisations of immunity. It analysed the ways in which immunity was marketed to the public in English-language Irish newspapers between 1890 and 1940, bringing digital humanities techniques to the medical humanities by blending the large scale afforded by digitised archives with an in-depth engagement with medical language and advancements. In this way, the article unpacked the understanding of immunity resulting from a consumer culture in which readers and shoppers were told that immunity could be found on the shelves of Irish shops, pharmacies, and showrooms. The rhetoric of the immune self is an inextricable part of the story of the modern Irish consumer, as the immune self was not a status conferred by a state, colonial or Free, but an identity created through purchasing power and the consumption of specific commodities, many of which were produced and marketed from outside the 32 counties. The immunity of the Irish consumer, therefore, was inevitably of a porous, shifting nature: local and foreign, public and private, natural and artificial, evoking risk and protection, of grave importance but often a simple everyday choice, as we see in Figure 4: ‘To keep yourself immune from chills/ From colds and all your other ills –/ “HAVE SHREDDED WHEAT FOR BREAKFAST”’.^144^

## Footnotes

1 William Boxwell, ‘A Sketch of the Development of Therapeutic Immunization’, *The Dublin Journal of Medical Science* 2 December 1912, 421–422.

2
*Ibid*., 422.

3 Richard Wellington Shegog, ‘Immunity and its Relation to Modern Preventative Medicine’, *The Dublin Journal of Medical Science* 1 April 1915, 293.

4
*Irish Independent* 28 October 1913, 3. Most advertisements were repeated in and across newspapers. In this article a citation is given for the earliest instance of the advertisement.

5
*Sunday Independent* 3 December 1916, 4.

6
*Irish Independent* 15 May 1929, 15.

7 Arthur M. Silverstein, *A History of Immunology* (San Diego: Academic Press, 1989); Warwick Anderson and Ian R. Mackay, *Intolerant Bodies: A Short History of Autoimmunity* (Baltimore: John Hopkins UP, 2014); Kenton Kroker, Jennifer Keelan and Pauline M. H. Mazumdar, eds, *Crafting Immunity: Working Histories of Clinical Immunology* (London: Routledge, 2016).

8 Ciara Breathnach, ‘Gender, Medicine and The State in Ireland, 1852–1922’, in Jyoti Atwal, Ciara Breathnach and Sarah-Anne Buckley, eds, *Gender and History: Ireland, 1852–1922* (London: Routledge, 2022), 78. See too Ruth Barrington, *Health, Medicine and Politics in Ireland 1900 to 1970* (Dublin: Institute of Public Administration, 1987).

9 See, for example, Tony Farmer, *Patients, Potions and Physicians: A Social History of Medicine in Ireland, 1654–2004* (Dublin: A. & A. Farmer, 2004).

10 B. N. Hedderman, *Glimpses of My Life in Aran: Some Experiences of a District Nurse in these Remote Islands, off the West Coast of Ireland* (Bristol: John Wright and Sons, 1917), 81, 95.

11 A short overview can be found in Diarmaid Ferriter, ‘Local government, public health and welfare in twentieth-century Ireland’, in Mary E. Daly, ed, *County and Town: One Hundred Years of Local Government in Ireland* (Dublin: Institute of Public Administration, 2001), 109–19.

12 For earlier contexts see James Kelly, ‘Health for Sale: Mountebanks, Doctors, Printers and the Supply of Medication in Eighteenth-century Ireland’, *Proceedings of the Royal Irish Academy: Archaeology, Culture, History, Literature* 2008, 108, 75–113.

13 See, for example, British Medical Association, *Secret Remedies: What they Cost and What They Contain* (London: British Medical Association, 1909); Anat Rosenberg, *The Rise of Mass Advertising: Law, Enchantment, and the Cultural Boundaries of British Modernity* (Oxford: Oxford University Press, 2022).

14 Lori Loeb, ‘Beating the Flu: Orthodox and Commercial Responses to Influenza in Britain, 1889–1919’, *Social History of Medicine*, 2005, 18.2, 203–224; Caitríona Foley, ‘“This Revived Old Plague”: Coping with Flu’, in Catherine Cox and Maria Luddy, eds, *Cultures of Care in Irish Medical History, 1750–1970* (Basingstoke: Palgrave Macmillan, 2010), 141–67.

15 For recent research that examines public ideas of immunity in a contemporary setting, see Mark Davis, *Selling Immunity Self, Culture and Economy in Healthcare and Medicine* (London, Routledge, 2022).

16 Lauren Kassell, ‘Paper Technologies, Digital Technologies: Working with Early Modern Medical Records’, in Anne Whitehead and Angela Woods, eds, *The Edinburgh Companion to the Critical Medical Humanities* (Edinburgh: Edinburgh University Press, 2016), 120–35; E. Thomas Ewing, Veronica Kimmerly, and Sinclair Ewing-Nelson, ‘Look Out for “La Grippe”: Using Digital Humanities Tools to Interpret Information Dissemination during the Russian Flu, 1889–90’, *Medical History*, 2016, 60.1, 129–31.

17 Mary E. Daly, ‘Death and Disease in Independent Ireland, c. 1920–1970: A Research Agenda’ in Catherine Cox and Maria Luddy, eds, *Cultures of Care in Irish Medical History, 1750–1970* (Basingstoke: Palgrave Macmillan, 2010), 230.

18 Greta Jones, ‘*Captain of all These Men of Death’: The History of Tuberculosis in 19th and 20th Century Ireland* (Amsterdam: Rudolphi, 2001), 2.

19 Hugh Oram, *The Advertising Book: The History of Advertising in Ireland* (Dublin: MO Books, 1986), 5.

20 ‘The Advertising Problem’, *Leabhar na hÉireann: The Irish Year Book* (Sinn Féin, Dublin, 1910), 309.

21 T. A. Grehan, ‘The Position of Advertising in Modern Commerce’, as reported in *The Evening Herald* 12 April 1911, 7.

22 Mary McCarthy, ‘The Changing Nature of Advertisements in The Freeman’s Journal: A Case Study, 1763–1924’, *Journal of Historical Research in Marketing*, 2014, 6.1, 151. See too John R. Strachan and Claire Nally, *Advertising, Literature and Print Culture in Ireland, 1891–1922* (London: Palgrave Macmillan, 2021); J. Fanning, ‘Branding and begorrah: the importance of Ireland’s nation brand image’, *Irish Marketing Review*, 2011, 21.1 and 2; Linda King and Elaine Sisson, eds, *Ireland, Design and Visual Culture: Negotiating Modernity, 1922–1992* (Cork: Cork University Press, 2011).

23 Bernadette Whelan, ‘Introduction to Irish Marketing History’, *Journal of Historical Research in Marketing*, 2014, 6.1, np.

24 Felix M. Larkin, ‘T.R. Larrington and the “new” Irish Independent, 1905–31’, in Mark O’Brien and Kevin Rafter, eds, *Independent Newspapers: A History* (Dublin: Four Courts Press, 2012), 30.

25 Stephanie Rains, *Advertising and Consumer Culture in Ireland, 1922–1962: Buy Irish* (Liverpool: Liverpool University Press, 2022), 18.

26
*Anglo-Celt*, 2 December 1916, 4.

27
*Limerick Leader*, 1 May 1905, 2.

28
*Nationalist and Leinster Times*, 24 May 1930, 3.

29
*Belfast Newsletter*, 6 June 1911, 5.

30
*Belfast Newsletter* 23 October 1928, 7.

31
*Nationalist and Leinster Times*, 14 September 1901, 3. This is a reference to the fact that cattle were often infected with bovine tuberculosis, as was milk, so many developed immunity to mycobacterium tuberculosis.

32
*Irish Press,* 31 December 1931, 3.

33 See, for example, *Skibbereen Eagle*, 17 June 1922, 6; *Evening Herald*, 19 February 1924, 2.

34
*Sunday Independent* 26 January 1908, 8.

35
*Leitrim Observer*, 20 January 1923, 4.

36
*Sunday Independent* 14 July 1907, 4. These germs included meningococcal meningitis. I am grateful to Ross Lawrenson for this point.

37
*Anglo-Celt*, 18 March 1911, 4.

38
*Westmeath Independent*, 25 August 1928, 8.

39
*Anglo-Celt*, 18 March 1911, 4.

40
*Irish Independent*, 24 July 1905, 7.

41
*Nenagh Guardian* 10 February 1923, 3.

42 Edmond J. McWeeny, ‘Immunity—I’, *The New Ireland Review*, July 1894, 287.

43
*Connaught Tribune* 8 September 1923, 4.

44 Ed Cohen, *A Body Worth Defending: Immunity, Biopolitics and the Apotheosis of the Modern Body* (London: Duke University Press, 2009), 4.

45
*The Irish Times*, 9 March 1928, 13.

46
*Irish Independent*, 14 December 1927, 4.

47
*Ballymena Observer*, 25 January 1935, 1.

48
*Irish Independent*, 12 November 1920, 1.

49
*The Irish Times*, 15 January 1929, 3.

50
*Leinster Express*, 10 November 1926, 8. Emphasis added.

51
*The Irish Times*, 7 March 1934, 5. Emphasis added.

52
*Derry Journal*, 19 May 1909, 4. Emphasis added.

53
*Irish Independent*, 13 January 1922, 3.

54
*Anglo-Celt*, 12 December 1931, 9.

55
*Irish Independent*, 5 February 1924, 4.

56
*Irish Independent*, 29 April 1924, 9.

57
*Weekly Irish Times*, 9 March 1929, 17.

58
*The Irish Times* 6 January 1937, 3.

59
*Waterford Standard* 12 December 1906, 4.

60
*Waterford Standard* 12 December 1906, 4.

61
*The Irish Times*, 27 February 1907, 10.

62
*Weekly Irish Times*, 9 October 1909, 18.

63
*Dublin Evening Telegraph,* 15 February 1915, 2.

64
*Fermanagh Times*, 24 June 1915, 1.

65
*Ibid.*

66
*The Irish Times*, 14 January 1932, 6.

67
*The Irish Times*, 11 January 1933, 4.

68
*The Irish Times* 6 February 1924, 7.

69
*Irish Independent*, 6 September 1929, 12.

70
*Irish Independent*, 20 September 1929, 6.

71 Congreve’s Elixir, *Sunday Independent*, 15 October 1911, 5.

72 Iglodine, *Irish Independent*, 25 March 1940, 1.

73 Phosferine nerve tonic, *Irish Independent*, 6 July 1909, 8.

74 Doan’s Backache Kidney Pills, *Anglo-Celt*, 7 December 1918, 7.

75 Bishop Varalettes, *The Irish Times* 6 February 1924, 7.

76 Atmos, *Irish Independent* 20 June 1932, 3.

77
*The Irish Times* 28 June 1938, 3.

78 William J. Thompson, ‘Mortality from Influenza in Ireland’, *Journal of the Statistical and Social Inquiry Society of Ireland,* 1920, 14.1, 1.

79 See, for example, Ida Milne, *Stacking the coffins: Influenza, War and Revolution in Ireland, 1918–19* (Manchester: Manchester University Press, 2018); Patricia Marsh, *The Spanish Flu in Ireland: A Socio-Economic Shock to Ireland, 1918–1919* (Basingstoke: Palgrave Macmillan, 2021).

80 For the advertising of patent medicines and cure-alls in Ireland during the pandemic see, for example, Caitriona Foley, *The Last Irish Plague: The Great Flu Epidemic in Ireland 1918–19* (Dublin: Irish Academic Press, 2011), 129–31.

81
*The Irish Times*, 5 March 1919, 3.

82
*Wicklow People*, 16 November 1918, 6.

83
*Londonderry Sentinel*, 3 December 1918, 2.

84 Thora Hands, *Drinking in Victorian and Edwardian Britain: Beyond the Spectacle of the Drunkard* (Cham: Palgrave Macmillan, 2018), 113. See too Amelia Bonea, Melissa Dickson, Sally Shuttleworth and Jennifer Wallis, *Anxious Times: Medicine and Modernity in Nineteenth-Century Britain* (Pittsburgh: University of Pittsburgh Press, 2019).

85
*Westmeath Examiner*, 24 November 1923, 7.

86 Hall’s Wine, *Irish Independent* 20 December 1912, 9.

87
*Irish Independent* 16 May 1906, 7.

88
*Sunday Independent* 10 March 1907, 8.

89 Caitríona Foley, ‘“This Revived Old Plague”: Coping with Flu’, in Catherine Cox and Maria Luddy, eds, *Cultures of Care in Irish Medical History, 1750–1970* (Basingstoke: Palgrave Macmillan, 2010), 157.

90
*Evening Irish Times*, 19 February 1909, 4.

91
*The Irish Times*, 25 February 1910, 5. Emphasis added.

92
*Irish Independent*, 19 April 1915, 1.

93
*The Irish Times*, 17 July 1922, 9.

94 Ovaltine advertisements made similar claims. See, for example, *Irish Independent*, 31 March 1921, 3. The Allenburys’ presentation of milk as immune was echoed by other companies selling dairy products. A year before the Clean Milk Campaign of 1926, Hughes Bros’ milk was described as strained by a scientific process which renders it ‘immune from contamination’ (*The Irish Times*, 11 June 1925, 4).

95
*Irish Independent*, 19 January 1915, 3.

96 McWeeny, ‘Immunity—I’. 287.

97 Wellington Shegog, ‘Immunity’, 299.

98 Deborah Brunton, *The Politics of Vaccination: Practice and Policy in England, Wales, Ireland, and Scotland, 1800–1874* (Boydell & Brewer, University of Rochester Press, 2008).

99 Jutta Kruse, ‘Saving Irish National Infants or Protecting the Infant Nation? Irish Anti-vaccination Discourse, 1900–1930’, *History Studies*, 2012, 13, 91–113.

100
*Dundalk Democrat*, 29 September 1917, 4.

101 For a detailed study on diphtheria and immunisation see Michael Dwyer, *Strangling Angel: Diphtheria and Childhood Immunization in Ireland* (Liverpool: Liverpool University Press, 2018).

102
*The Irish Times*, 6 January 1937, 3.

103
*Irish Press*, 16 February 1938, 6.

104
*Irish Press*, 13 July 1940, 5.

105 Felix M. Larkin, ‘T.R. Larrington and the “new” Irish Independent, 1905–31’, in Mark O’Brien and Kevin Rafter, eds, *Independent Newspapers: A History* (Dublin: Four Courts Press, 2012), 30; Ian Kenneally, ‘The Irish Independent and the Irish Revolution’, in Mark O’Brien and Kevin Rafter, eds, *Independent Newspapers: A History* (Dublin: Four Courts Press, 2012), 45; Fearghal McGarry, ‘Irish Newspapers and the Spanish Civil War’, *Irish Historical Studies*, 2022, 33.129, 68–90.

106 Scott’s Emulsion, *The Irish Times*, 6 November 1902, 3.

107
*The Irish Times* 6 Jan 1937, 3.

108
*Sunday Independent* 21 January 1923, 7; *The Irish Times*, 19 June 1923, 9; *Irish Independent* 6 July 1909, 8; *The Irish Times* 29 April 1924, 7.

109
*Irish Independent* 5 February 1924, 4.

110 The Irish Times 5 March 1919, 3. This advertisement was repeated in newspapers such as *The Sketch, Western Mail, Yorkshire Post and Leeds Intelligencer, Nottingham Evening Post* and *The Scotsman* in February and March 1919.

111
*Freeman’s Journal*, 21 August 1896, 3.

112 Belfast Telegraph, 7 March 1907, 2.

113 Pat Oats, *Belfast Newsletter*, 21 February 1922, 6. See too White’s Wafer Oats, Belfast Telegraph 11 February 1902, 3.

114 For example, Denis Murphy in Doneraile (*Limerick Leader*, 15 April 1910, 2); James Cashen in Birr (*Nenagh Guardian*, 5 March 1921, 2); Ernest W. Parker in Kilkenny (*Kilkenny People*, 18 March 1922, 1); Michael Telvin and Sons in Kells (*Anglo-Celt*, 16 October 1926, 6); J. C. Morgan in Dunleer (*Dundalk Democrat*, 26 October 1929, 13); James H. North and Co. in Dublin (*The Irish Times* 2 October 1937, 16).

115
*Lisburn Standard*, 29 March 1918, 2.

116
*Irish Independent*, 29 July 1907, 1.

117 O’Keeffe’s, *The Nationalist*, 7 November 1908, 6.

118
*The Irish Times*, 14 March 1927, 7.

119 Susan Sontag, *Illness as Metaphor and AIDS and Its Metaphors* (London: Macmillan, 2001); Emily Martin, ‘Toward an Anthropology of Immunology: The Body as Nation State’, *Medical Anthropology Quarterly*, 1990, 4.4, 410–426.

120
*The Irish Times*, 29 January 1900, 3.

121 See, for example, Freeman’s Journal, 20 December 1890, 8, *The Irish Times*, 7 December 1901, 12; *Meath Chronicle*, 24 January 1903, 5.

122
*The Irish Times*, 8 December 1906, 12.

123 Pesco, *The Irish Times*, 30 November 1926, 3.

124 Dexter’s Weatherproof coats, *The Irish Times* 23 September 1913, 4. Cebedene made a similar promise of immunity for those wearing their trench overcoat. *Irish Independent*, 27 December 1924, 1.

125 Celia Marshik, ‘The Modern(ist) Mackintosh’, *Modernism/modernity*, 2012, 19.1, 43–71.

126 Dryfoot Boots, *Ulster Herald*, 9 November 1935, 10.

127
*Westmeath Examiner*, 25 March 1899, 4.

128
*Belfast Weekly Telegraph*, 12 April 1919, 6.

129
*The Irish Times*, 7 March 1938, 5.

130
*Belfast Newsletter*, 27 April 1906, 2. An earlier version, with typos, appeared in *Western People*, 17 February 1906, 4.

131
*The Irish Times*, 3 May 1911, 4.

132
*The Irish Times*, 25 September 1924, 9.

133 Dunlop Tyres, *Evening Irish Times*, 23 March 1910, 4; Swift Bikes, *Belfast Newsletter*, 19 June 1903, 1; Avon Rubber, *The Irish Times*, 25 July 1922, 3.

134 J. P. Tracy, *The Irish Times*, 15 July 1926, 2.

135 Raleigh, *Connaught Telegraph*, 17 July 1915, 6.

136
*The Irish Times*, 8 April 1901, 1.

137
*Freeman’s Journal*, 8 June 1896, 8.

138
*Irish Independent*, 5 June 1920, 2.

139 India Super Tyres, *The Irish Times* 7 Aug 1930, 15. Emphasis added.

140 John Fergus (T. A. Grehan), ‘Advertising. The News Element’, *Irish Independent* 2 April 1909, 4. See too Colum Kenny, ‘Tom Grehan: Advertising Pioneer and Newspaper Man’, in Mark O’Brien and Kevin Rafter, eds, *Independent Newspapers: A History* (Dublin: Four Courts Press, 2012), 52–66.

141
*Irish Press*, 5 October 1934, 8.

142
*Irish Press*, 7 June 1934, 9.

143 Emily Martin, *Flexible Bodies: Tracking Immunity in American Culture—from the Days of Polio to the Age of AIDS* (Boston: Beacon Press, 1994); Nik Brown, *Immunitary Life: Biopolitics of Immunity* (London: Palgrave Macmillan, 2019); Eula Biss, *On Immunity: An Inoculation* (Minneapolis: Graywolf Press, 2014); Roberto Esposito, ‘Community, Immunity, Biopolitics’, *Angelaki: Journal of the Theoretical Humanities*, 2013, 18, 83–90.

144
*Irish Press*, 16 December 1932, 2.

## Notes

**Maebh Long** is a Senior Lecturer at the University of Waikato. She is the author of *Assembling Flann O’Brien* (2014), the editor of *The Collected Letters of Flann O’Brien* (2018) and the co-editor of *New Oceania: Modernisms and Modernities in the Pacific* (2019). Maebh is currently leading a project, funded by the Royal Society of New Zealand, which examines the ways ‘immunity’ became a contagious metaphor during the early twentieth century.

